# Facilitation or hindrance? The impact of downward social comparison on adversarial growth

**DOI:** 10.3389/fpsyg.2024.1307393

**Published:** 2024-06-20

**Authors:** Ting Nie, Jing Wu, Qiao Yan

**Affiliations:** ^1^School of Business, Macau University of Science and Technology, Taipa, Macao SAR, China; ^2^School of Business, Zhuhai College of Science and Technology, Zhuhai, Guangdong, China

**Keywords:** downward social comparison, self-acceptance, gratitude, adversarial growth, interpersonal sensitivity

## Abstract

**Introduction:**

While adversity can bring great challenges to individuals' life and work, many people also find ways to make positive changes and adapt to these difficult circumstances. Individuals tend to make social comparisons more frequently and intensely when faced with adversity or high stress. The study attempts to examine the influence mechanism of downward social comparison on individual adversarial growth.

**Methods:**

By collecting data from 353 Chinese who have experienced adversities in the past 3 years, the study validates the dual mediating model of gratitude and self-acceptance and explores the moderating effect of interpersonal sensitivity.

**Results:**

The findings indicate that: downward social comparison can increase the occurrence of adversarial growth by enhancing individuals' self-acceptance and gratitude. Compared to individuals with low interpersonal sensitivity, individuals with high interpersonal sensitivity are more likely to facilitate the occurrence of adversarial growth through self-acceptance and gratitude due to downward social comparison.

**Discussion:**

In the face of adversity, individuals can regain self-confidence and respond positively by comparing themselves to people in worse situations than themselves. In particular, individuals with higher interpersonal sensitivity are more likely to benefit from downward social comparisons and grow from adversity.

## 1 Introduction

Over the life course, adversities are inevitable, but personal responses and changes after adversity may vary significantly. Some will suffer from depression and anxiety, which can lead to negative behaviors including post-traumatic stress disorder, addiction, withdrawal, or violence (Brandell and Ringel, 2019; Infurna et al., 2023). Others may view adversities as a valuable life experience that results in positive changes: new philosophies, improved relationships, and enhanced skills that can help achieve greater success later in life (Tedeschi and Calhoun, 1996; Joseph and Linley, 2006). Especially in the past 3 years, the world has been overwhelmed by the epidemic, and many people's lives and work have been challenged. They may have to face some major changes such as unemployment, illness, loss of family members, and divorce. Individuals can grow from adversity by thinking about its meaning carefully and responding to it in a positive way. The study of adversarial growth is more valuable in the Post-Epidemic Era.

Previous studies have mainly explained the occurrence of adversarial growth in terms of available social support and coping strategies. When individuals experience adversity, they are more likely to recover and strengthen themselves quickly if they can get emotional and financial support from family members, friends, and organizations (Carr, 2019). And as compared to negative coping strategies such as complaining, denial, and self-blame, positive approaches such as acceptance, planning, mindfulness, and humor are more helpful for individuals to develop adversarial growth (Joseph, 2009; Farhadi et al., 2022). It is commonly understood that adversity can come as a sudden shock to anyone, placing them in a state of high stress. As social beings, individuals often compare themselves to others who have experienced similar situations and seek possible references to solve problems. The tendency to make social comparisons usually increases temporarily during times of stress or change (Buunk et al., 2005). The role of social comparison is consistently controversial (Taylor and Lobel, 1989). Upward social comparison can set a direction and example for individuals to move forward (Gürel et al., 2020), but also has the potential to trigger jealousy and anxiety in individuals (Van de Ven, 2017). Downward social comparison can alleviate individuals' anxiety (Wills, 1981), but it tends to trigger a mapping effect that may lead individuals to give up their efforts (Smith, 2000). In studies on trauma, many scholars support the significant positive effects of upward social comparison based on Western samples, which can motivate individuals to respond to difficult situations and recover quickly. Success cases and role models can inspire hope and encouragement in people experiencing trauma, giving them the confidence to overcome obstacles and rebuild their lives. Upward social comparisons can provide a reference for individuals in their pursuit of self-development and positive growth, which has been widely validated and recognized in existing research. However, the influence of downward social comparison on those who experience adversity lacks more elaborate exploration and empirical investigation.

As the Selective Accessibility Model explains, individuals will compare themselves with selected standards and form similarity or dissimilarity judgments. Once this perception is formed, the individual will continue to reinforce this self-evaluation and consequently choose subsequent behaviors (Mussweiler, 2003, 2020). In the face of adversity, individuals may exhibit cognition or behaviors that are different from their normal state. Their tendency toward self-denial is more likely to lead to fear comparisons with others, especially with those who are better than they are, which can cause them to further lose confidence and give up coping (McCarthy and Morina, 2020; Wiesche et al., 2024). Adversity experiences may lead to damaged self-perceptions and a blow to self-esteem (Joseph and Linley, 2006). In this case, upward social comparison is more likely to make individuals perceive differences from successful personnel (Vogel et al., 2020). The dissimilarity assumption may motivate individuals to pay more attention to the successes and achievements of others and solidify the gap perception with others. Whereas in downward social comparisons, similar adversity situations are more likely to trigger empathy. Individuals may seek to comfort and alleviate negative emotions. The similarity assumption may trigger individuals to value more the encouragement and experience they receive from individuals who are also going through difficult situations. They are more likely to find their value and meaning through scrutiny and reflection, thus rebuilding their internal identity (Mussweiler, 2003; Vogel et al., 2020). For individuals experiencing adversity, downward social comparisons are also valuable, and their influence mechanisms and boundary conditions on individual adversarial growth are worth further exploration.

Social Comparison Theory states that individuals need to maintain stable and accurate self-evaluations by comparing themselves to others (Buunk and Gibbons, 2007). When individuals are in adversity, downward social comparisons can lead to a clearer awareness of the strengths they have by comparing themselves to those who are less successful or have disadvantages than they are. They are also likely to reflect on and appreciate the resources and opportunities they have (Nicuţǎ and Constantin, 2021). In line with Social Cognitive Theory, when individuals realize that the opportunities they have are highly valued relative to others, such reflection can help promote greater satisfaction and acceptance of their current situation (Bandura et al., 1986). Furthermore, individuals can learn from others who are also in a state of adversity. Acceptance and gratitude for the status quo can increase individuals' motivation to pursue new opportunities and changes. To restore themselves to their original state, individuals may try to use the resources they have to resolve and respond to this threatened situation (Wood et al., 2009). These efforts can prevent individuals from depression due to adversity and are more likely to result in positive changes (McCarthy and Morina, 2020). For individuals experiencing adversity, downward social comparison enables individuals to gain emotional comfort and cognitive self-acceptance, which is more likely to trigger adversarial growth.

Social comparisons are more frequent when individuals face higher levels of stress and uncertainty. China is a relationship-oriented society in which individuals value interpersonal interactions and strive to maintain harmonious relationships with others. Therefore, Chinese interpersonal bonds are usually stronger and individuals also have higher interpersonal sensitivity (Chen et al., 2020; Lei et al., 2021). People are more sensitive to social feedback, more alert to others' reactions, and more attentive to the behaviors and opinions of those around them (Boyce and Parker, 1989). When individuals experience adversities, they also hold higher sensitivity to the emotions and behaviors of others, and the effect of social comparison is amplified by their interpersonal sensitivity. For individuals with high interpersonal sensitivity, self-acceptance and gratitude resulting from downward social comparison are more likely to facilitate the occurrence of adversarial growth. The value of downward social comparison may therefore be more salient for individuals with high interpersonal sensitivity.

Based on Social Cognitive Theory and Selective Accessibility Model, the study attempts to explain the influence mechanism of downward social comparison on individual adversarial growth by collecting data from 353 individuals in China who have experienced adversities in the past three years. We validate a dual mediating model of gratitude and self-acceptance and explore the moderating effect of interpersonal sensitivity. The exploration and validation of the influence mechanisms and boundary conditions of downward social comparison on individual growth under adversity conditions complements existing research on trauma and adversity. Meanwhile, on the practical side, the study may also provide valuable suggestions for individuals coping with adversity and trauma recovery.

## 2 Literature review and research hypotheses

### 2.1 Downward social comparison and adversarial growth

Social comparison is the process by which people compare their abilities, feelings, situations, and opinions with others in real life. People need to assess their abilities and perspectives. When there is a lack of objective criteria in a situation, they will satisfy the need for self-assessment by comparing themselves to others. This process of comparing one's state with that of others to obtain a clear self-assessment is known as social comparison (Festinger, 1954). It is a pervasive psychological phenomenon in human social life that reflects an individual's sensitivity to information and ideas related to other people (Gibbons and Buunk, 1999). Regarding the direction of comparison, there are three types of social comparisons: parallel, downward, and upward. Parallel social comparisons are comparisons with similar people, while downward and upward social comparisons are comparisons with people who are worse and better than they are, respectively.

Close interpersonal relationships are more likely to trigger individuals' spontaneous tendency to make social comparisons (Ekström and Hjort, 2009). During upward social comparison, individuals experience two critical emotions: negative jealousy and positive admiration (Van de Ven, 2017). It can increase an individual's sense of self-efficacy to witness the success of others. Individuals are motivated and inspired by those who are superior when they believe they can achieve similar achievements (Lockwood and Kunda, 1997; Gürel et al., 2020). Admiration leads to imitation, which in turn enhances one's performance (Rheu et al., 2023). At the same time, individuals may also feel that their self-esteem is threatened. Based on the intrinsic need to maintain self-worth, they may move away from or even devalue the comparator (Stapel and Suls, 2004). When individuals' abilities and perspectives are externally challenged, their motivation to sustain or improve self-esteem drives them to make downward social comparisons. By comparing with targets inferior to themselves, they can maintain subjective well-being and positive self-evaluation (Hakmiller, 1966). It is also possible at times for individuals to experience high anxiety and decreased self-esteem as a result of mapping poor situations onto themselves when downward social comparisons are made (Smith, 2000; McCarthy and Morina, 2020). Neither upward nor downward social comparisons necessarily lead to positive or negative effects; the specific effects depend on the context (Gilbertson and Graves, 2023).

When individuals experience adversity, it does not necessarily result in severe traumatic reactions and the consequences may vary (Shigemoto et al., 2016). Some people will generate adversarial growth as a response to positive psychological changes due to the traumatic event, including enhanced personal relationships, increased competence, reorientation within the self, and changes in life philosophy (Tedeschi and Calhoun, 1996; Joseph and Linley, 2006). Meanwhile, their ability to handle crises and their self-confidence is strengthened, and they usually believe they are capable of facing future difficulties (Tedeschi and Calhoun, 1996). Adversarial growth is a common occurrence and is likely to happen in individuals who experience various adversities. Approximately 35.75% of those experiencing adversities report growth in some way (Parikh et al., 2015). A survey conducted on victims of rape reveals that 25% of the respondents reported improved relationships with family members they were previously estranged from Burt and Katz (1987). While struggling with an adversarial event, individuals may rethink the meaning of their lives, develop closer connections with their families, and rebuild their belief systems (Aldwin, 2007). As a result, they will increase psychological coping resources, alleviate negative emotions, and reduce the emergence of post-traumatic stress disorder (Janoff-Bulman, 2010). Existing research has confirmed that many factors can predict the occurrence of adversarial growth. An individual's cultural literacy is positively correlated with adversarial growth, and a high level of education can help avoid negative psychological reactions caused by adversity and trauma (Joseph et al., 2004). Support from family and friends can help individuals have the courage to face stress and seek proactive changes, and growth in adversity is more evident (Carr, 2019). Individuals with higher psychological resilience tend to view perceived risks and adversities as challenges. They will continue to learn and grow their skills as they cope with difficulties (Maguen et al., 2006; Yeung et al., 2022). Positive coping strategies are also significantly associated with adversarial growth. Mindfulness, humor, religious support, help-seeking and so on can assist individuals in confronting difficult situations, coping more effectively with anxiety, and developing healthy thinking patterns (Joseph and Linley, 2006; Farhadi et al., 2022).

### 2.2 The mediating role of self-acceptance

Self-acceptance is the attitude that an individual has toward his or her worth and ability, which is a major manifestation of a healthy personality (Carson and Langer, 2006). It emphasizes the individual's complete acceptance of themselves, regardless of whether their words and actions are correct, wise, or appropriate, and regardless of whether others approve, respect, or love them (Ellis, 1977). Hayes et al. (2011) identified self-acceptance as a cognitive model in which individuals maintain an independent self as well as a flexible approach to self-experience. It greatly empowers individuals to be cognitively and behaviorally adaptive. Social support, peer recognition, and family parenting are all significantly related to individual self-acceptance (Fatima et al., 2018; Rolle-Whatley, 2021). Increased control perception can also enhance an individual's self-approval and intrinsic motivation to complete the task (Floricica and Tasente, 2022). Self-acceptance and interpersonal relationships influence each other. Individuals with high self-acceptance are more confident and comfortable in interpersonal interactions and have significantly lower social anxiety. They can perceive their traits, and recognize and respect themselves. In contrast, those who do not accept themselves dislike their personalities and lack self-confidence (Aykut Ceyhan and Ceyhan, 2011). Self-acceptance is also an important indicator of psychological well-being, and individuals with high self-acceptance usually have positive feelings about themselves as well as their past experiences (Zhou and Xu, 2019). In emotional terms, recognition of the self and one's life is the main source and most salient feature of happiness. They perceive more support from society and less hostility toward others (Fernández et al., 2015). While individuals who do not approve and censure themselves tend to treat others with similar attitudes. People with low self-acceptance typically view others more negatively and are less tolerant of others, which negatively affects their quality of interpersonal relationships (Kornyeyeva and Boehnke, 2013).

Social comparison emphasizes the use of others as a contrast for self-evaluation in the absence of objectivity (Buunk et al., 2001). In the process of comparison, individuals tend to reconceptualize and reposition themselves. As indicated in Selective Accessibility Model, individuals engage in comparing themselves to specific standards and then form judgments of similarity or dissimilarity. Once this perception is established, individuals tend to reinforce this self-evaluation and consequently make subsequent behavioral choices (Mussweiler, 2003). When facing negative life events, individuals are more likely to develop helplessness and self-denial (Gambaro et al., 2020). At this point, comparing themselves to people who are in a worse situation or similar situation can help them maintain emotional stability by protecting their self-esteem and self-worth (Wills, 1981; Stewart et al., 2013). Downward social comparison decreases an individual's reference system for self-evaluation, and it is a well-adapted mechanism for coping with stressful events and maintaining psychological wellbeing (Buunk et al., 2001). Individuals' confidence in coping with adversity is also boosted when they realize that others who are worse off are actively trying to change the situation (Nicuţǎ and Constantin, 2021). With the aim of self-enhancement, maintaining self-esteem, or regulating emotions (Gerber et al., 2018), individuals tend to have higher acceptance of their present state and gain a good sense of themselves (Gentile et al., 2020). Acceptance of negative situations is often accompanied by higher self-efficacy and better psychosomatic health, which can buffer the negative effects of stress and anxiety (Murakami and Latner, 2015; Sansinenea et al., 2020). Self-acceptance does not exclude change, and it is seen as the starting point for self-transcendence (Bernard, 2014). Downward social comparison can reduce their anxiety, weaken their isolation, and help them regain the confidence to cope with adversities. By enhancing individuals' self-acceptance and satisfying their intrinsic motivation for self-enhancement, the downward social comparison may result in adversarial growth. Hypothesis 1 is proposed:

*H1: Self-acceptance mediates the relationship between downward social comparison and individual adversarial growth*.

### 2.3 The mediating role of gratitude

Gratitude emerges when an individual receives assistance or support that generates a positive and pleasant emotional experience (McCullough et al., 2002). Grateful people will show appreciation for the benefits they receive and also would like to give back in return (Soscia, 2007). Gratitude can be considered both as a personality trait and as a dynamic state. As a personality trait, it can differ from person to person. On the other hand, gratitude can also be seen as a temporary and external state that arises in response to a particular situation or behavior (Eamons and Mc Cullough, 2003). In a highly dynamic social environment, a positive and lasting positive relationship can be established between individuals due to gratitude (Rusk et al., 2016). Grateful individuals are more optimistic and better at finding the good in life. They have a higher sense of well-being and are more likely to feel happiness and satisfaction (McCullough et al., 2002; Gilbertson and Graves, 2023). Gratitude facilitates the transformation of positive emotions into positive behaviors. Individuals with high gratitude may express positive responses and feedback. They often have an optimistic view of the past and future and would like to adopt positive coping strategies in adversities (Watkins et al., 2003; Layous et al., 2023). If people are grateful for support and kindness from others, it generally triggers positive reciprocity. This interaction between giver and recipient is beneficial in promoting harmonious interpersonal relationships and significantly predicts the occurrence of their pro-social behaviors (Williams and Bartlett, 2015).

In line with Social Cognitive Theory, an individual's behavior is determined by the perception and interpretation of environmental stimuli. By observing and imitating the behavior of others, individuals can acquire new skills and continuously adjust their self-efficacy (Bandura et al., 1986). Social comparisons allow individuals to reflect on themselves and shift their attention toward positive aspects of life (Vogel et al., 2020). In particular, downward social comparisons help individuals recognize their strengths and redirect their focus toward appreciating what they already possess, rather than being completely immersed in anxiety about their hardships (Burt and Katz, 1987). It is more capable of inspiring a state of gratitude in individuals. In the face of adversity, they can maintain hope for the future and confidence in changing the current situation (Nicuţǎ and Constantin, 2021). The cultivation of gratitude enables individuals to bounce back swiftly from setbacks and fosters a positive outlook toward their future. They will experience fewer negative emotions such as hopelessness and depression (Karadag, 2009; Gilbertson and Graves, 2023). By adopting an attitude of gratitude, individuals are more willing to actively learn from others and value the support they may receive. This openness enables individuals to broaden their skills, gain new perspectives, and develop creative strategies to overcome existing dilemmas (Gentile et al., 2020). Gratitude enhances an individual's psychological resilience and is an important predictor of post-traumatic growth (Wood et al., 2009). As a result of engaging in downward social comparisons, individuals are more inclined to feel content and appreciative of their current situation. In turn, they exhibit increased initiative in managing challenging circumstances and tend to expand their knowledge and skills through personal reflection and learning. Hypothesis 2 is proposed:

*H2: Gratitude mediates the relationship between downward social comparison and individual adversarial growth*.

### 2.4 The moderating role of interpersonal sensitivity

Interpersonal sensitivity, as a stable personality, reflects an individual's sensitivity to perceive and react to the emotions and behaviors of others. Characteristics of interpersonal sensitivity include sensitivity to social feedback, alertness to the reactions of others, and excessive attention to the behaviors and assertions of others. They are often overly sensitive to and fearful of perceived or actual criticism or rejection (Boyce and Parker, 1989). Due to their insecurity, negative cognitive patterns, and sense of being controlled, individuals with high interpersonal sensitivity are prone to self-depreciation and self-doubt. They are less confident during social interactions, so they often minimize the risk of rejection by constantly adjusting their behaviors (Marin and Miller, 2013; Costa et al., 2015). Interpersonal sensitivity is significantly correlated with depression and anxiety. High interpersonal sensitivity is more likely to generate poor interpersonal relationships, academic burnout, lower subjective wellbeing, and addictive behaviors (Chow et al., 2013; Stavropoulos et al., 2022). Current research generally confirms that interpersonal sensitivity is the result of both genetic and environmental influences. Highly protective parenting styles can predispose children to high interpersonal sensitivity. Supportive and caring parents can reduce the development of this trait. The influence of mothers is generally stronger than that of fathers (Doise and Palmonari, 2010; Smorti et al., 2022). Individuals' interpersonal sensitivity varies significantly by demographic characteristics such as age and gender. As individuals grow, they become more confident due to stable interpersonal relationships and their interpersonal sensitivity generally tends to decrease (Wilhelm et al., 2004). Women also generally have higher interpersonal sensitivity than men, who will place more value on maintaining good relationships with others (Otani et al., 2008). Furthermore, in the workplace, leadership style and power can significantly affect individuals' interpersonal sensitivity. Egocentric leaders are relatively less interpersonal sensitive (Schmid Mast et al., 2009). Most studies point out that high-powered people tend to see things from their own perspective, and they are less likely to care about and respond to others' perceptions. Power increases their social distance, which reduces their motivation to concern others and weakens their interpersonal sensitivity (Magee and Smith, 2013). Low-powered individuals need to pay more attention to information details, so they exert more effort on noticing others, and interpersonal sensitivity increases accordingly (Kossowska et al., 2016).

Individuals with high interpersonal sensitivity are more likely to perceive problems as threatening and unsolvable. They have lower self-efficacy in coping with difficult situations (Boyce and Parker, 1989). When confronted with stressful events, they often employ avoidance or rejection coping strategies (Wilhelm et al., 2004; Masillo et al., 2012). Adversity environments are always accompanied by increased uncertainty and risk. As identified in Selective Accessibility Model, individuals who form similar assumptions in social comparisons will further reinforce this perception through their behaviors (Mussweiler, 2003). Individuals with high interpersonal sensitivity experience more pronounced anxiety in adversity due to their alertness and sensitivity to criticism, rejection, and interpersonal conflict. Their need for recognition and belonging is usually stronger (Slanbekova et al., 2019). The choices and performance of similarly situated individuals may have a more pronounced effect on them. At this stage, downward social comparison is more likely to stimulate individual reflection and regain confidence. They will realize that they are not alone and that adversity is a situation that many people face (McCarthy and Morina, 2020). Engaging in downward social comparisons reduces individuals' inclination to deny themselves. Acceptance serves as the first step toward initiating changes (Bernard, 2014). Hypotheses 3 and 4 are proposed:

*H3: Interpersonal sensitivity positively moderates the relationship between downward social comparison and self-acceptance*.
*H4: Interpersonal sensitivity positively moderates the mediating effect of self-acceptance between downward social comparison and individual adversarial growth*.

Individuals with high interpersonal sensitivity are particularly concerned about their relationships with others, and are constantly alert to the behaviors and emotions of others. They may change their behaviors to meet the expectations of others (Harb et al., 2002). Adversity may make individuals feel more uneasy about all aspects of interpersonal relationships. Individuals who are highly sensitive to interpersonal dynamics frequently seek reassurance and validation through positive interactions with others. They possess a strong concern for how others perceive them and often harbor a fear of criticism and rejection (Boyce and Parker, 1989). Downward social comparison reduces anxiety, enhances self-satisfaction, and is more likely to perceive kindness from others. Gratitude as a positive psychological state helps individuals recover from adversity (Karadag, 2009) and maintain good relationships with others. Such benign interactions are even more valuable for individuals with high interpersonal sensitivity. They are more likely to form empathy with individuals in similar or worse situations and value all that they have (Black et al., 2022). They may be more willing to initiate learning and positive change due to high gratitude. Interpersonal sensitivity contributes to enhancing the impact of downward social comparison on individual adversarial growth through gratitude. Hypotheses 5 and 6 are proposed:

*H5: Interpersonal sensitivity positively moderates the relationship between downward social comparison and gratitude*.
*H6: Interpersonal sensitivity positively moderates the mediating effect of gratitude between downward social comparison and individual adversarial growth*.

## 3 Methods

### 3.1 Participants and procedure

This study tries to explain the influence mechanism of downward social comparison on individual adversarial growth and the moderating effect of interpersonal sensitivity by collecting data from 353 Chinese who have experienced adversities in the past three years. The study was reviewed by the Research Ethics Committee of Business School at Macau University of Science and Technology. All methods in the study were performed following the Declaration of Helsinki. Respondents were informed of the purpose and procedures of the study before starting the survey. All data collected in the study were used only for research purposes. Respondents participated in the survey on a voluntary basis and had the right to withdraw at any time. All questionnaires without complete answers were considered invalid.

This study selected those who had experienced adversities since the outbreak of the COVID-19 Epidemic as respondents. Adversities are the difficulties and setbacks that individuals experience in their lives. This study defined adversity as a significant negative life event with reference to the Social Readjustment Rating Scale (Holmes and Rahe, 1967). We identified the top 10 negative events as individual adversities as follows: widowhood, divorce, separation, incarceration, death of a family member, unemployment, sexual disorders, illness, debt, and demotion. Respondents were required to complete the questionnaire based on whether they had experienced these adversities in the past three years. The survey was conducted online through Wenjuanxing.com, a major online survey platform in China. To reduce the effect of common method bias, data in this study were collected in two phases with one-month interval (Podsakoff et al., 2003). Time 1: questionnaires were distributed through convenience sampling to measure downward social comparison, gratitude, and interpersonal sensitivity. 779 respondents participated the survey, and 473 indicated that they had these experiences in the past three years and 455 valid questionnaires were returned, with a valid return rate of 96.2%. Time 2: questionnaires were distributed to the same group of participants by matching telephone numbers to measure self-acceptance, adversarial growth, and demographic characteristics. 353 valid questionnaires were returned, with a valid return rate of 77.6%.

In terms of gender, males accounted for 43.9% (155) of respondents, females accounted for 56.1% (198) of respondents, and female respondents were slightly more than male respondents. In terms of age, individuals less 20 years old accounted for 1.1% (4) of respondents, individuals between 21 to 25 years old accounted for 34.6% (122) of respondents, individuals between 26 to 30 years old accounted for 14.7% (52) of respondents, individuals between 31 to 40 years old accounted for 19.8% (70) of respondents, individuals between 41 to 50 years old accounted for 15 % (53) of respondents, individuals between 51 to 60 years old accounted for 9.3% (33) of respondents, and individuals more than 60 years old accounted for 5.4% (10) of respondents. The age distribution of respondents was relatively balanced. The majority of respondents have received higher education, and 73.4% (259) of respondents had bachelor's degrees or higher. In terms of marital status, 49.3% (174) of the respondents were married.

### 3.2 Measures

Self-report scales were used in the study and measured with Likert 5-point scales from “completely disagree” to “completely agree.” Spss 26, Amos 24, and Process 3.4 software were used for data analysis.

#### 3.2.1 Downward social comparison

Downward social comparison is a comparison with people who are worse than they are (Gibbons and Buunk, 1999). Downward social comparison was measured with a 6-item scale developed by Gibbons and Buunk (1999), e.g., “I sometimes compare myself to people who are in worse situations than I am.” In this study, Cronbach's alpha coefficient of this scale was 0.851.

#### 3.2.2 Self-acceptance

Self-acceptance is the attitude that an individual has toward his or her personal worth and ability, which emphasizes the individual's complete acceptance of themselves (Ellis, 1977). Self-acceptance was measured with a 16-item scale developed by Cong and Gao (1999), e.g., “Overall, I am satisfied with myself.” In this study, Cronbach's alpha coefficient of this scale was 0.727.

#### 3.2.3 Gratitude

Gratitude is a positive and pleasant emotional state when an individual receives help or support from others (McCullough et al., 2002). Gratitude was measured with a 6-item scale developed by McCullough et al. (2002), e.g., “I feel grateful for so many things in my life.” In this study, Cronbach's alpha coefficient of this scale was 0.787.

#### 3.2.4 Adversarial growth

Adversarial growth is positive psychological changes after experiencing adversities, including enhanced personal relationships, increased competence, reorientation within the self, and changes in life philosophy (Joseph and Linley, 2006). Adversarial growth was measured with a 10-item scale developed by Cann et al. (2010). e.g., “After adversity, I have learned what is more important in life.” In this study, Cronbach's alpha coefficient of this scale was 0.892.

#### 3.2.5 Interpersonal sensitivity

Interpersonal sensitivity reflects an individual's sensitivity to perceive and react to the emotions and behaviors of others (Boyce and Parker, 1989). Interpersonal sensitivity was measured with a 7-item scale developed by Boyce and Parker (1989), e.g., “I'm worried about what others think of me.” In this study, Cronbach's alpha coefficient of this scale was 0.884.

#### 3.2.6 Control variables

Gender, age, education, and marriage were considered as control variables. The classification is as follows: gender (male; female); age (less than 20; 21–25; 26–30; 31–40; 41–50; 51–60; more than 60); education (high school; bachelor; master; doctor); Marriage (single; married).

## 4 Results

### 4.1 Common method bias test

First of all, Harman's single-factor test and confirmatory factor analysis are used to test common method bias in the study (Podsakoff et al., 2003). In Harman's single-factor test, the variance explained by the first factor is 33.83%, which is much lower than the threshold level of 50%. In confirmatory factor analysis, the model fit of the five-factor model (downward social comparison, self-acceptance, gratitude, adversarial growth, interpersonal sensitivity) is acceptable (χ^2^/*df* = 1.765, TFI = 0.946, CFI = 0.956, GFI = 0.903, RMSEA = 0.043, and RMR = 0.034), which is better than other alternative models. The model fit of the single-factor model is far from acceptable (χ^2^/*df* = 7.951, TFI = 0.388, CFI = 0.431, IFI = 0.502, RMSEA = 0.144, and RMR = 0.109). The common method bias in the study is not a big concern.

### 4.2 Descriptive statistics and correlation analysis

The results of descriptive statistics and correlation analysis are shown in Table 1. When controlling the effects of gender, age, education, and marriage, downward social comparison is significantly associated with self-acceptance (0.436, *p* < 0.01), gratitude (0.237, *p* < 0.01), and adversarial growth (0.246, *p* < 0.01). Self-acceptance is significantly associated with adversarial growth (0.377, *p* < 0.01). Gratitude is significantly associated with adversarial growth (0.268, *p* < 0.01). In addition, interpersonal sensitivity is also significantly associated with downward social comparison (–0.117, *p* < 0.01), self-acceptance (–0.163, *p* < 0.01), and adversarial growth (–0.350, *p* < 0.01). Preliminary correlation analysis results support the research hypotheses.

**Table 1:** Mean, standard deviation and correlation statistics (*n* = 353).

	**Mean**	**SD**	**1**	**2**	**3**	**4**	**5**	**6**	**7**	**8**
Gender	1.53	0.497								
Age	3.63	1.584	0.054							
Edu	2.00	0.796	-0.025^**^	-0.280^**^					
Mar	1.49	0.501	-0.041	0.756^**^	-0.275^**^					
DSC	3.86	0.627	-0.027	-0.128^*^	0.247^**^	-0.077				
SA	3.64	0.580	-0.125^*^	-0.116^*^	0.112^*^	-0.061	0.436^**^			
GA	3.60	0.528	-0.048	0.113^**^	0.003	0.066	0.237^**^	0.212^**^		
AG	3.73	0.528	-0.142^**^	0.028	0.118^**^	0.070	0.246^**^	0.377^**^	0.268^**^	
IS	3.42	0.387	0.217^**^	-0.048	-0.084	-0.135^**^	-0.117^**^	-0.163^**^	0.006	-0.350^**^

^**^p < 0.01, ^*^p < 0.05; DSC, Downward Social Comparison; SA, Self-acceptance; GA, Gratitude; AG, Adversarial Growth; IS, Interpersonal Sensitivity.

### 4.3 Hypothesis testing

Model 2, Model 3, and Model 5 validate the mediating effect of self-acceptance between downward social comparison and individual adversarial growth with the approach suggested by Baron and Kenny (1986). As shown in Table 2, when controlling the effects of gender, age, education, and marriage, downward social comparison has a significant positive relationship with self-acceptance (0.428, *p* < 0.001) and adversarial growth (0.230, *p* < 0.001). When simultaneously considering the effects of downward social comparison and self-acceptance on adversarial growth, self-acceptance has a significant positive relationship with adversarial growth (0.325, *p* < 0.001). The influence of downward social comparison on adversarial growth is not significant (0.091, *p* > 0.05). The hierarchical regression analysis results show that the effect of downward social comparison on adversarial growth is fully mediated by self-acceptance. Hypothesis 1 is supported.

**Table 2:** Hierarchical regression of mediating effect (*n* = 353).

	**M1**	**M2**	**M3**	**M4**	**M5**	**M6**	**M7**	**M8**
	**AG**	**AG**	**SA**	**GA**	**AG**	**AG**	**SA**	**GA**
Gender	-0.133^*^	-0.131^*^	-0.109^*^	-0.043	-0.095	-0.141^*^	-0.086	0.039
Age	-0.006	0.022	-0.073	-0.178^*^	0.046	-0.019	-0.092	0.160
Edu	0.143^*^	0.089	-0.012	-0.026^*^	0.093	0.095	-0.029	-0.29
Mar	0.108	0.090	0.019	-0.054	0.084	0.102	0.007	-0.043
DSC		0.230^***^	0.428^***^	0.263^***^	0.091	0.169^***^	0.371^***^	0.233^***^
SA					0.325^***^			
GA						0.230^***^		
IS			.				-0.088	0.037
DSC*IS			.				0.242^***^	0.168^***^
*R* ^2^	0.043	0.092	0.206	0.081	0.176	0.141	0.271	0.109
F	3.926^**^	7.069^***^	18.010^***^	6.116^***^	12.358^***^	9.740^***^	18.299^***^	6.005^***^

^***^p < 0.001, ^**^p < 0.01, ^*^p < 0.05; DSC, Downward Social Comparison; SA, Self-acceptance; GA, Gratitude; AG, Adversarial Growth; IS, Interpersonal Sensitivity.

Model 2, Model 4, and Model 6 validate the mediating effect of gratitude between downward social comparison and individual adversarial growth. As shown in Table 2, when controlling the effects of gender, age, education, and marriage, downward social comparison has a significant positive relationship with gratitude (0.263, *p* < 0.001) and adversarial growth (0.230, *p* < 0.001). When simultaneously considering the effects of downward social comparison and gratitude on adversarial growth, gratitude has a significant positive relationship with adversarial growth (0.230, *p* < 0.001). The influence of downward social comparison on adversarial growth has decreased (0.169, *p* < 0.001). The hierarchical regression analysis results show that the effect of downward social comparison on adversarial growth is partially mediated by gratitude. Hypothesis 2 is supported.

Model 7 validates the moderating effect of interpersonal sensitivity between downward social comparison and self-acceptance. As shown in Table 2, when controlling the effects of gender, age, education, and marriage, the moderating effect of interpersonal sensitivity between downward social comparison and self-acceptance is significant (0.242, *p* < 0.001). For individuals with high interpersonal sensitivity, the impact of downward social comparison on self-acceptance is stronger. Hypothesis 3 is supported.

Model 8 validates the moderating effect of interpersonal sensitivity between downward social comparison and gratitude. As shown in Table 2, when controlling the effects of gender, age, education, and marriage, the moderating effect of interpersonal sensitivity between downward social comparison and gratitude is significant (0.168, *p* < 0.001). For individuals with high interpersonal sensitivity, the impact of downward social comparison on gratitude is stronger. Hypothesis 4 is supported.

When simultaneously considering the mediating effect of self-acceptance and gratitude between downward social comparisons and individual adversarial growth, the specific results are shown in Table 3. The estimated value of the total direct effect is (0.057, 0.046) and the 95% confidence interval is [–0.034, 0.147]. The confidence interval includes zero, which indicates that the direct effect is not significant in the dual mediating model. The estimated value of the total indirect effect is (0.150, 0.053), and the 95% confidence interval is [0.759, 0.266]. The confidence interval excludes zero, which indicates that the indirect effect is significant in the dual mediating model. The estimated value of the indirect effect of self-acceptance is (0.113, 0.043) and the 95% confidence interval is [0.039, 0.204]. The confidence interval excludes zero, which indicates that the mediating effect of self-acceptance is significant. hypothesis 1 is further supported. The estimated value of the indirect effect of gratitude is (0.037, 0.017) and the 95% confidence interval is [0.009, 0.075]. The confidence interval excludes zero, which indicates that the mediating effect of gratitude is significant. hypothesis 2 is further supported. The estimated value of indirect effect diffidence is (0.076, 0.038) and the 95% confidence interval is [0.007, 0.156]. The confidence interval excludes zero, which means the mediating effect of self-acceptance is stronger than that of gratitude. The dual mediating effect between downward social comparison and individual adversarial growth is supported.

**Table 3:** Dual mediating effect (*n* = 353).

	**Effect**	**SE**	**LLCI**	**ULCI**
Direct effect	0.057	0.046	-0.034	0.147
Total indirect effect	0.150	0.053	0.759	0.266
Indirect effect (SA)	0.113	0.043	0.039	0.204
Indirect effect (GA)	0.037	0.017	0.009	0.075
Diff (SA-GA)	0.076	0.038	0.007	0.156

SA, Self-acceptance; GA, Gratitude; LLCI, Low level of 95% confidence interval; ULCI, Upper level of 95% confidence interval.

The results of the moderated mediating effect validation are shown in Table 4. Model 1 tests whether interpersonal sensitivity positively moderates the mediating effect of self-acceptance between downward social comparison and adversarial growth. The 95% confidence interval of moderated mediating effect (0.135 0.048) is [0.040, 0.229], and the interval tested in bootstrapping excludes zero, which means interpersonal sensitivity positively moderates the relationship between downward social comparison and individual adversarial growth through self-acceptance. For individuals with high interpersonal sensitivity, the impact of downward social comparison on individual adversarial growth through self-acceptance is more significant. Hypothesis 5 is supported. Model 2 tests whether interpersonal sensitivity positively moderates the mediating effect of gratitude between downward social comparison and adversarial growth. The 95% confidence interval of moderated mediating effect (0.067, 0.035) is [0.005, 0.141], and the interval tested in bootstrapping excludes zero, which means interpersonal sensitivity positively moderates the relationship between downward social comparison and individual adversarial growth through gratitude. For individuals with high interpersonal sensitivity, the impact of downward social comparison on individual adversarial growth through gratitude is more significant. Hypothesis 6 is supported.

**Table 4:** The moderated mediating effect (*n* = 353).

	**Moderating effect**						**Moderated mediating effect**				
**Variables**		**Effect**	**SE**	**p**	**LLCI**	**ULCI**		**Index**	**SE**	**LLCI**	**ULCI**
M1	Int	0.443	0.089	0.000	0.269	0.628	W1	0.135	0.048	0.040	0.229
SA	L	0.177	0.061	0.004	0.058	0.343	L	0.054	0.028	0.009	0.118
	H	0.520	0.050	0.000	0.421	0.618	H	0.158	0.048	0.064	0.249
M2	Int	0.302	0.090	0.001	0.126	0.478	W1	0.067	0.035	0.005	0.141
GA	L	0.057	0.061	0.350	-0.063	0.178	L	0.013	0.015	–0.020	0.039
	H	0.291	0.053	0.000	0.191	0.390	H	0.065	0.025	0.016	0.114

SA, Self-acceptance; GA, Gratitude; LLCI, Low level of 95% confidence interval; ULCI, Upper level of 95% confidence interval.

## 5 Discussions and conclusions

**Figure 1:**
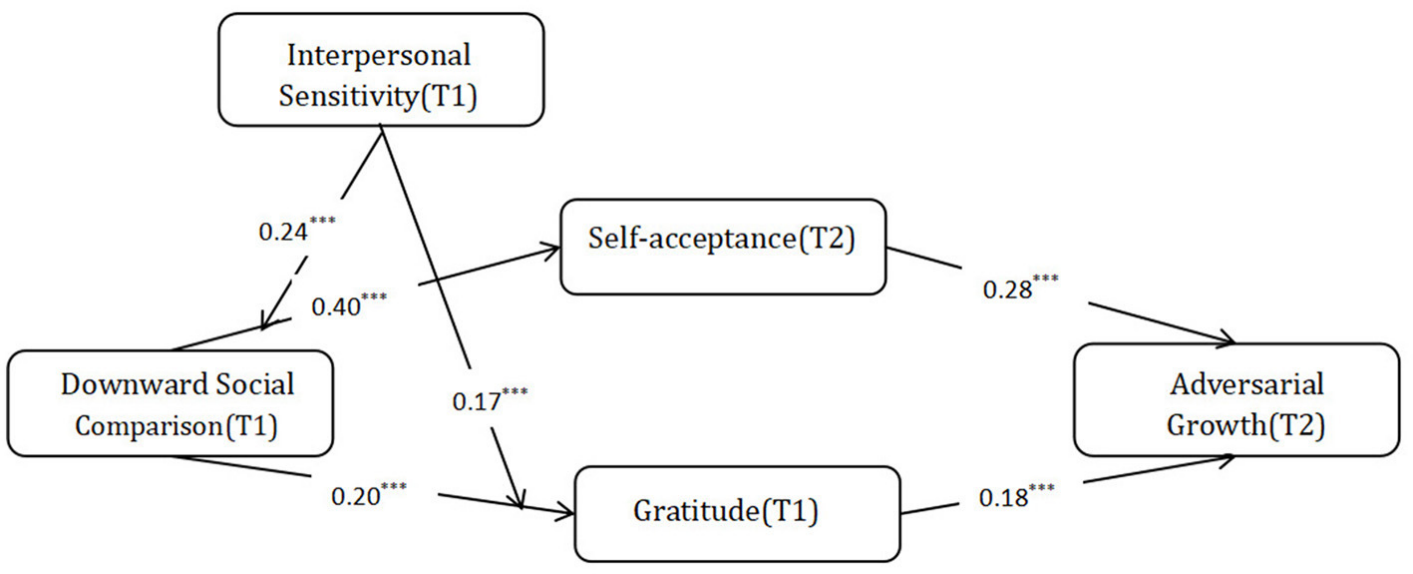
Theoretical framework. ***p < 0.001.

Adversity brings great challenges to an individual's life, but many people can also grow through adversity by increasing their skills, improving their relationships, identifying new opportunities, and establishing a new philosophy of life in the coping process (Joseph, 2009). Through a statistical survey of 353 individuals who experienced adversities in the past three years, this study verified that self-acceptance and gratitude played a mediating role between downward social comparison and individual adversarial growth, and formed a double mediating model. Interpersonal sensitivity positively moderated the indirect effect of downward social comparison on individual adversarial growth through self-acceptance and gratitude. Compared to low interpersonal sensitivity individuals, individuals with high interpersonal sensitivity were more likely to facilitate the occurrence of adversarial growth through self-acceptance and gratitude due to downward social comparison. All research hypotheses were supported as shown in Figure 1.

### 5.1 Theoretical implications

Downward social comparison can increase the occurrence of adversarial growth by enhancing individuals' self-acceptance and gratitude. This finding validates the explanation of the Selective Accessibility Model for the social comparison mechanism. The individual will compare oneself to selected standards, forming judgments of similarity or dissimilarity. Once this cognitive process is established, the individual will constantly reinforce this self-evaluation. While upward social comparisons can stimulate people's sense of competition and motivation, prompting them to strive for higher goals and better achievements (Lockwood and Kunda, 1997; Gürel et al., 2020). Previous research has indicated that when individuals experience negative life events, they may question their perspectives and abilities (Gambaro et al., 2020). Compared with people who are inferior to themselves can protect one's self-worth to some extent and maintain emotional stability. The positive effects of downward social comparison are especially evident when individuals' abilities and perspectives are challenged by outside sources (Wills, 1981). it not only satisfies an individual's intrinsic motivation to maintain or increase self-esteem but also facilitates the individual's subjective wellbeing and positive self-evaluation (Stewart et al., 2013). By observing the positive attitudes and behaviors of individuals with similar experiences, individuals are more likely to move beyond feelings of helplessness. They may believe that they can also change their situation by taking the initiative, as many individuals in similar circumstances have done. This study provides further confirmation that individuals who experience adversity can use downward social comparison to alleviate psychological stress and prevent self-denial (Gambaro et al., 2020; McCarthy and Morina, 2020). By comparing themselves to those who are worse off, individuals can develop a greater appreciation for what they have, regain their motivation, and effectively deal with challenges. Downward social comparison serves as an adaptive mechanism for regulating an individual's mood and enhancing their self-esteem (Smith, 2000). It helps individuals to gain a good sense of self and enhance their willingness to self-reinforce (Gerber et al., 2018), which drives individuals' improvement and growth. When experiencing adversity, downward social comparison is more likely to trigger positive change through individual self-acceptance and gratitude.

Most previous research has explained the causes and conditions of adversarial growth in terms of social support and coping styles (Carr, 2019; Ali et al., 2023). Individual traits and needs can also greatly influence the underlying mechanisms that shape adversarial growth. China is typically a relationship-oriented society where people expect to maintain good and relatively intimate relationships with others. Individuals are more sensitive to the reactions and behaviors of others. Close interpersonal relationships tend to trigger spontaneous social comparison tendencies (Ekström and Hjort, 2009). In particular, when people are in periods of stress or facing change, their willingness to social comparison also temporarily increases (Buunk et al., 2005). Social comparison is the basis for individual interpersonal interactions and behavioral choices. In line with Social Cognitive Theory, individuals acquire information and learn by observing others. They will tend to reshape their behavior in reflection (Bandura et al., 1986). Individuals with high interpersonal sensitivity care more about the results of social comparisons, which is consistent with the findings of previous research (Exline and Lobel, 1999). The role of social comparison is thus amplified. They are not only hypersensitive and fearful of “perceived or actual criticism or rejection” (Bianchi et al., 2015), but are also extraordinarily concerned about their relationships. They are constantly alert to other behaviors and emotions and may even change their behavior to meet the expectations of others (Harb et al., 2002). Individuals with high interpersonal sensitivity are more likely to experience positive outcomes from engaging in downward social comparisons. Comparisons with individuals who are perceived as inferior can lead to increased self-evaluation and satisfaction (Pan and Peña, 2020). They will have more confidence to learn from others, strive to change the status quo, and achieve personal growth when faced with challenges. It is also a complement to the study on the formative boundary of adversarial growth.

### 5.2 Practical implications

Everyone encounters challenges throughout their lives, particularly in the past three years when the COVID-19 pandemic caused significant disruptions to people's lives and work. Adversities such as illness, loss of loved ones, and unemployment have become prevalent concerns for some people. Those who have experienced them hope to recover quickly and to achieve possible growth from these adversities.

First, when experiencing adversity, the positive effects of downward social comparison are more pronounced than upward social comparison. Individuals can compare themselves more often with those who are inferior to them to maintain self-esteem and obtain positive self-evaluation. Although upward social comparison can be used to achieve self-motivation and progress by identifying the gap with excellent people. But for individuals in adversity, they are often faced with a life blow and the first thing to address is recovery from self-denial. Upward social comparison tends to trigger high anxiety and self-abandonment, while downward social comparison helps individuals re-accept themselves and value what they have. In times of adversity, comparing oneself with individuals who are in relatively worse situations is more helpful for individuals to recover and initiate change.

Second, individuals should change their mindset and view adversity as an opportunity for personal development. While adversity is often accompanied by loss and grief, it also allows individuals to take re-examination of their lives and work. Especially through the downward social comparison, individuals should be able to realize that compared with many people, they already have a lot of things to cherish. Even in the face of adversity, there are still many people who have not given up hope and are continuing to work hard. Learning from others and seeking ways for improvement is the beginning of gaining personal growth. In the future, individuals need to love themselves more and also be grateful for what they have. While seeking emotional support from friends and family, they should also actively utilize preventive measures to identify potential risks and avoid repeating mistakes (Cao and Liu, 2022). Growth can be achieved through positive changes in thought and behaviors.

Finally, for individuals with high interpersonal sensitivity, the positive effects of downward social comparison are more pronounced. In times of adversity, individuals who are more sensitive to the behaviors and reactions of others are inclined to engage in spontaneous social comparison more frequently. They are also more likely to benefit from downward social comparison. Their feelings of gratitude and self-acceptance will serve as powerful motivators for initiating and embracing positive changes. When individuals experience adversity, the tendency toward self-denial may cause them to reject upward social comparisons. They can feel more comfort and satisfaction when encouraged to make comparisons with others in similar or worse situations. In particular, individuals with high interpersonal sensitivity should be motivated to learn more about the positive attitudes and coping strategies of others who have also experienced adversity. This can help them regain their confidence (Pan and Peña, 2020). Through self-reflection and learning from others, they are more likely to achieve positive changes.

## 6 Limitations and future study

There are certain limitations in this study. First of all, the respondents in this study were all from China with culture characterized by relationship orientation and collectivism. Individuals are more sensitive to interpersonal relationships. They try to maintain harmonious relationships and strive to make a good impression on others. At the same time, the phenomenon of social comparison is more common in China. This is a significant difference from the situation in many countries, so the findings of this study may have some external validity issues. In future studies, cross-cultural comparative studies may be considered to better explain the mechanisms of social comparison on individual adversarial growth in different contexts. Self-report scales were used in this study. We tried to reduce the influence of common method bias through a two-stage survey and statistical control. In future studies, it may be beneficial to incorporate experimental, case study, interview, and multiple methods to gather additional evidence and confirm the underlying mechanism through which social comparison influences an individual's adversarial growth. Furthermore, the respondents in this study had a higher proportion of females and most of them have received higher education, which is not fully congruent with the current distribution of demographic characteristics in China. Are there gender and educational differences in individuals' coping strategies in the face of adversity? Is there a gender preference between upward social comparison and downward social comparison? Does education level predict individual adversity growth? Adversity is bound to be encountered by every person in life, and addressing it effectively requires taking into account individual variations and employing suitable approaches to accelerate recovery and generate positive change. The differential influence of demographic characteristics on individual adversarial growth mechanisms can be further verified in future studies. Moreover, The respondents in this study were individuals who had experienced severe adversity during the epidemic. Specific experiences and perceptions may have made them different from the general population in terms of social comparison tendencies. This study focused only on the impact of downward social comparison. Future research could also explore differences in the functioning mechanisms of the two social comparisons across time for individuals. By measuring the gap between an individual and their comparator, the impact of this gap on an individual's cognition and behavior can be examined, further validating the Selective Accessibility Model. Finally, the investigation in this study was conducted at the end of the COVID-19 epidemic, which greatly affected individuals' daily lives and work routines. The entire social atmosphere was in an unprecedented state, and people's psychological well-being may have been compromised. The shock of the pandemic may amplify the frustration and insecurity that individuals may feel in the face of adversity. The high-pressure state of society as a whole may also influence individuals' judgment of the status quo and alter personal coping strategies. Further validation is needed to confirm whether the findings of this study are consistent across regular environments. Apart from investigating the individual antecedents and consequences of adversarial growth, future studies could also shed light on the influence of social, community, and organizational factors by conducting cross-level analysis.

## Data availability statement

The raw data supporting the conclusions of this article will be made available by the authors, without undue reservation.

## Ethics statement

The studies involving humans were approved by Research Ethics Committee of Business School at Macau University of Science and Technology. The studies were conducted in accordance with the local legislation and institutional requirements. The participants provided their written informed consent to participate in this study.

## Author contributions

TN: Conceptualization, Formal analysis, Methodology, Resources, Software, Supervision, Visualization, Writing – original draft, Writing – review & editing. JW: Investigation, Writing – review & editing. QY: Data curation, Investigation, Visualization, Writing – original draft, Writing – review & editing.
